# Role of Mononuclear Cardiomyocytes in Cardiac Turnover and Regeneration

**DOI:** 10.1007/s11886-020-01289-y

**Published:** 2020-05-19

**Authors:** Cora Becker, Michael Hesse

**Affiliations:** Institute of Physiology I, Life & Brain Center, Medical Faculty, University of Bonn, Bonn, Germany

**Keywords:** Cardiac regeneration, Cardiomyocyte turnover, Cardiomyocyte ploidy, Mononuclear

## Abstract

**Purpose of Review:**

The typical remodeling process after cardiac injury is scarring and compensatory hypertrophy. The limited regeneration potential of the adult heart is thought to be due to the post-mitotic status of postnatal cardiomyocytes, which are mostly binucleated and/or polyploid. Nevertheless, there is evidence for cardiomyocyte turnover in the adult heart. The purpose of this review is to describe the recent findings regarding the proliferative potential of mononuclear cardiomyocytes and to evaluate their function in cardiac turnover and disease.

**Recent Findings:**

There is overwhelming evidence from carbon-dating in humans and multi-isotope imaging mass spectrometry in mice that there is a very low but detectable level of turnover of cardiomyocytes in the heart. The source of this renewal is not clear, but recent evidence points to a population of mononuclear, diploid cardiomyocytes that are still capable of authentic cell division. Controversy arises when their role in cardiac repair is considered, as some studies claim that they contribute to repair by cell division while other studies do not find evidence for hyperplasia but hypertrophy. Stimulation of the mononuclear cardiomyocyte population has been proposed as a therapeutic strategy in cardiac disease.

**Summary:**

The studies reviewed here agree on the existence of a low annual cardiomyocyte turnover rate which can be attributed to the proliferation of mononuclear cardiomyocytes. Potential roles of mononucleated cardiomyocytes in cardiac repair after injury are discussed.

## Introduction

Cardiovascular diseases are the main cause of death in the industrial world. As the mammalian heart displays only limited regenerative potential, new therapeutic strategies for cardiac repair are urgently needed. Mammalian hearts consist of mononuclear and multinuclear cardiomyocytes, but their functional differences are still unknown.

Shortly after birth, the mammalian hearts switches from hyperplastic growth by cardiomyocyte division to hypertrophic growth by an increase of individual cardiomyocyte size. During this transition, cardiomyocytes undergo variations in the cell cycle such as acytokinetic mitosis (karyokinesis, but no cytokinesis), resulting in a binuclear cell and endoreduplication (no karyokinesis or cytokinesis), resulting in a polyploid cell. In mice, the fraction of binuclear cardiomyocytes reaches around 90% by postnatal day 14 [1, 2] and the fraction of polyploid nuclei is ~ 45% [3]. In the human heart, polyploidy predominates with ~ 66%, while binuclear cardiomyocytes make up ~ 25% of all cardiomyocytes [4, 5]. The biological function of the different cardiomyocyte populations is still unclear. So far, polyploidy in the heart has been related mostly to hypertrophy [6, 7].

Cell cycle activity in cardiomyocytes decreases during early postnatal heart growth and in the adult mammalian heart, it is undetectable or very sparse. 3H-thymidine incorporation studies in mice came to the conclusion that 0.0005% of all cardiomyocytes are in the cell cycle [8]. Assuming that all cell cycle active cardiomyocytes will eventually divide an annual proliferation rate of 1.09% was calculated [9]. The idea of postnatal proliferation in human hearts was sparked by the group of Jonas Frisén [10]. In this intriguing study, which was based on radiocarbon dating, the human heart was found to replace 39% of its cardiomyocytes during a lifespan of 75 years. However, the source of the new cardiomyocytes could not be determined by the study and the calculated annual turnover rate decreased with age to 0.3% at 70 years. This poor proliferative potential of adult cardiomyocytes could explain the limited regenerative response after cardiac lesions. In contrast to this, it has been shown many times that hearts from adult zebrafish as well as amphibians such as newts are able to regenerate after cardiac injury [11, 12]. As a source for this remarkable potential, resident cardiomyocytes were identified [13]. In mammals, neonatal mice seem to have increased regenerative capacity, too. But there is still a controversy to which extent regeneration takes place [14, 15]. Nevertheless, this potential is lost after the first week in life and adult mammalian hearts do not display significant regeneration after experimentally induced damage [16]. Apparently, one major difference between cardiomyocytes in adult zebrafish and mammals is the degree of nucleation. The fish heart consists almost exclusively of mononuclear, diploid cardiomyocytes [17••], while mammalian hearts display a large proportion of polyploid and multinucleated cardiomyocytes. Strikingly, induction of either binucleation or polyploidy in fish cardiomyocytes massively impairs its regenerative capacity [18, 19]. Also in neonatal mice, the proportion of mononuclear, diploid cardiomyocytes is very high and this could explain the regenerative potential after induction of a cardiac injury [16, 20]. According to this, it was apparent to attribute the regenerative capacity to this cardiomyocyte population.

## Proliferative Potential of Mononucleated Cardiomyocytes

Two phenomena involving cell cycle activity have been described: a replacement of cardiomyocytes causing an annual turnover and activation of cardiomyocytes in the border zone after a cardiac lesion. As a source for both, it has been suggested that mononuclear, diploid cardiomyocytes are still able to re-enter the cell cycle and divide. The first publication demonstrating mononuclear cardiomyocyte division in mice used periostin as a stimulator [21]. Furthermore, the growth factor neuregulin1 was found to stimulate cell cycle activity and division in mononuclear cardiomyocytes [22]. In both studies, neither the ploidy of cardiomyocytes nor a basic cardiac turnover was examined and there was no cardiomyocyte division reported in the non-stimulated control groups, but merely cell cycle activity.

The connection of mononuclear, diploid cardiomyocytes and proliferative response during cardiac repair was first proposed in a study in mice using multi-isotope imaging mass spectrometry with ^15^N-thymidine incorporation as a marker for newly synthesized DNA and a transgenic lineage tracing model [23]. This study convincingly demonstrated that newly formed cardiomyocytes were mononuclear and diploid and that they were derived from pre-existing cardiomyocytes. Also in this study, cardiomyocyte annual turnover was attributed to this cell population and determined as being 0.76%, which is similar to the rate found by Loren Fields group [9]. Interestingly, after experimentally induced cardiac injury, reactivation of cell cycle activities leads to polyploidy and hypertrophy and remarkably, increased cell division in the border zone (3.2%). However, the evidence provided for authentic cell division and proliferation of mononucleated, diploid cardiomyocytes was indirect and considered to be the only remaining explanation based on the data obtained. The method of multi-isotope imaging mass spectrometry was further implemented to study cardiac adaptation after endurance exercise. Two months of running exercise induced formation of 4.6-fold more mononucleated, diploid cardiomyocytes compared to sedentary mice [24]. Additionally, exercise provided a beneficial effect for coping with cardiac injury by inducing cardiomyocyte proliferation in the border zone of myocardial infarction.

As the most likely source of the observed cardiomyogenesis are mononucleated, diploid cardiomyocytes, a more detailed analysis of these population was the next logical step. The proportion of mononuclear, diploid cardiomyocytes seems to vary highly among mouse inbred strains [18]. Those with a higher proportion of mononuclear cardiomyocytes have been claimed to functionally recover better after myocardial infarction as compared to strains with a low proportion, and this was attributed to proliferation and increase in numbers of mononuclear, diploid cardiomyocytes [18]. These cells were characterized as being smaller than the other mononuclear cardiomyocytes and made up approximately half of the total mononuclear cardiomyocyte population. Transcriptome analysis of good versus poor regenerating mouse strains revealed the kinase Tnni3k to be one determinant of the proportion of mononuclear diploid cardiomyocytes. Unfortunately, annual turnover was not addressed in this study and again evidence for cardiomyocyte division was only indirect.

A very intriguing concept was proposed after comparing the proportion of diploid mononuclear cardiomyocytes in different species from zebrafish with more than 95% to humans and mice with less than 10% [25]. The authors found a correlation between metabolic rate, body temperature, and proportion of mononuclear diploid cardiomyocytes and demonstrated an influence of thyroid hormones on cardiac regeneration. In cardiomyocyte-specific thyroid receptor-alpha knockout mice, heart weight, cardiomyocyte number, and proportion of mononuclear, diploid cardiomyocytes increased significantly. Also, cardiac regeneration after myocardial infarction was improved due to proliferation of mononucleated diploid cardiomyocytes. The conclusion was made that the acquisition of endothermy, which is regulated by thyroid hormones, could have impaired the cardiac regenerative potential. Further experiments need to be performed to prove this with, e.g., the silky anteater which has a fraction of around 60% mononuclear diploid cardiomyocytes and should, therefore, have a remarkable regenerative capacity.

Further studies characterized a sub-population of mononucleated cardiomyocytes, leading to the discovery of small, hypoxic mononuclear cardiomyocytes which were claimed to be the source of an annual cardiac turnover of 0.3–1% as evidenced by fate mapping [26]. The identified population was very small (0.051 + − 0.0075% of total cardiomyocytes) and its DNA content was not determined. Given the extremely small percentage of this population of cardiomyocytes, they are likely a distinct population or a unique subpopulation of mononuclear cardiomyocytes from the ones described in the aforementioned studies. Additional experiments will be needed to confirm the role of this subpopulation. Taken together, numerous studies to date have reported the presence of mononuclear cardiomyocyte populations that display cell cycle activity and these populations may represent the source of cardiomyocyte turnover in the adult heart.

What discriminates a mononuclear from a binuclear cardiomyocyte? It is unknown if the two nuclei in a binucleated cardiomyocyte have the same transcriptional activity as the sole nucleus in a mononuclear cardiomyocyte or if one nucleus is inactive or expresses only a subset of the cellular mRNAs. By using a transgenic mouse model, it has been shown that transcription of several genes is different in the two nuclei of a binuclear cardiomyocyte [27]. However, it is not clear to what extent these differences persist and if there are epigenetic differences such as changes in the methylation pattern or chromosomal superstructure. As single cell and single nuclear transcriptional and epigenetic tools are now available to address these questions, it will be intriguing to see in future studies what exactly is the unique role of an individual nucleus in a multinucleated cardiomyocyte and how this influences proliferation and regeneration.

## Limits of the Proliferative Potential of Mononuclear Cardiomyocytes

While the evidence for the existence of a mononuclear, diploid population of cardiomyocytes in the adult heart is quite robust, there is no consensus on their functional role and especially if they are still able to undergo authentic cell division after cardiac lesion. Even in the extensively studied mouse model of neonatal heart regeneration, it is not entirely clear if the observed myogenesis is exclusively caused by hyperplasia [16] or if there is a hypertrophic component caused by accelerated binucleation [28]. Most likely both phenomena are taking place simultaneously and participate in the cardiac regeneration process.

In adult mouse hearts, there are several lines of evidence against the possibility of significant proliferation of mononuclear, diploid cardiomyocytes after myocardial injury: Postnatal cardiomyocytes display a split-phenotype of their centrosome (loss of cohesion and separation of the two centrioles) which are essential components of the spindle apparatus and therefore necessary for cell division [29]. According to this finding, only cells with intact centrosomes are able to undergo regular cell division while those with a split-phenotype can only undergo variants of the cell cycle such as endoreduplication or binucleation. Interestingly, the authors could not find any cardiomyocytes in adult mouse hearts that had intact centrosomes, thereby implicating that mononuclear, diploid cardiomyocytes are affected by the split phenotype, too.

Another reason for the impairment of proliferative potential of adult cardiomyocytes is the rapid loss of telomere ends during postnatal heart growth [30•]. In turn, telomere shortening leads to binucleation, as it increases the chance of the formation of DNA bridges that impair cell division. It remains unanswered in this study if mononuclear, diploid cardiomyocytes are not affected by telomere shortening and could, therefore, bypass the block in cytokinesis.

A pro-proliferative mononuclear cardiomyocyte population should have a distinct transcriptome when compared to other cardiomyocytes. This was addressed in a recent study of the transcriptional profile of mononuclear versus binuclear cardiomyocytes from adult mouse hearts analyzed by single-cell RNA-seq. In such a setting, it should be easy to find a still regenerative population of mononuclear, diploid cardiomyocytes which is distinct from other cardiomyocytes. Surprisingly, there was no difference reported when it came to differentially expressed genes. Ploidy was not addressed in this study, but an unbiased analysis of 2767 cardiomyocytes of which 715 were mononucleated should be sufficient to detect the diploid population. Additional experiments need to be performed in a cardiac injury setting as this might be the trigger for the mononuclear, diploid cardiomyocytes to proliferate. On the other hand, such a scenario seems to be very unlikely. It was claimed that acute cardiac injury is a trigger for mononuclear, diploid cardiomyocytes to proliferate and to repair the injury to some degree which in turn leads to a better outcome. However, the degree of cell cycle induction in cardiomyocytes after cardiac injury is under debate as multiple studies have found only neglectable activation. Studies using ^3^H-*thymidine* labeling reported a basic cell cycle activity in 0.0006% of all cardiomyocytes which increased to 0.0083% after cardiac injury [8]. This was confirmed by our study using a transgenic reporter mouse overexpressing a GFP-tagged version of the midbody protein anillin [31]. In this study, cell cycle activity in cardiomyocytes after cardiac injury was attributed entirely to endoreduplication. Studies with human hearts after myocardial infarction came to the same conclusion [7, 32]. While the presence and extent of cardiomyocyte division after injury will continue to be actively investigated, it is clear that the level of cell division in adult cardiomyocytes is quite low (no more than 5% of mononuclear, diploid adult cardiomyocytes exhibit cell cycle activity) and insufficient to compensate for the massive loss of cardiomyocytes after infarction.

In neonatal mice, genetic lineage tracing demonstrated an exclusive contribution of pre-existing cardiomyocytes to heart growth [33]. There was no significant increase in clonal cardiomyocyte expansion observed following myocardial infarction, demonstrating the limited capability of cardiomyocytes to proliferate during cardiac repair. However, ploidy was not addressed in this study and the origin of the lineage-traced cardiomyocyte population was not identified. Lastly, cellular metabolism is a critical process that must be assessed in any cardiomyocyte population claimed to represent a pro-proliferative population. A metabolic switch in all cardiomyocytes from glycolysis to fatty acid oxidation takes place shortly after birth to enable maturation. This coincides with a decrease in cardiomyocyte cell cycle activity which leads to binucleation and endoreplication [34]. Studies are ongoing to actively assess whether driving a change in metabolic status will lead to direct cardiomyocyte division or variants of cell cycle activation such as endoreplication or binucleation. In conclusion, multiple studies have described several impediments excluding a significantly increased proliferation of mononuclear, diploid cardiomyocytes after cardiac injury.

## Conclusion/Outlook

From the current evidence, it is not clear if there is a special role of mononuclear, diploid cardiomyocytes during cardiac repair following myocardial infarction. This is due to a lack of direct evidence for cell division of these cardiomyocytes. Methods used so far such as genetic lineage tracing or even stereology provide only indirect evidence of authentic cell division. Ideally, identification of genetically labeled mononuclear, diploid cardiomyocytes undergoing division in a heart section or live imaging of dividing cardiomyocytes in a Langendorff heart using dual-photon microscopy would ultimately demonstrate the claimed cell division potential of mononuclear, diploid cardiomyocytes. Furthermore, it is mandatory to show that there is no split phenotype of the centrosomes and no shortage of telomeres and chromosome fusion events in these cells. As long as these points remain unaddressed, the claim that mononuclear, diploid adult cardiomyocytes are able to undergo authentic cell division should remain speculative.

**Fig. 1:**
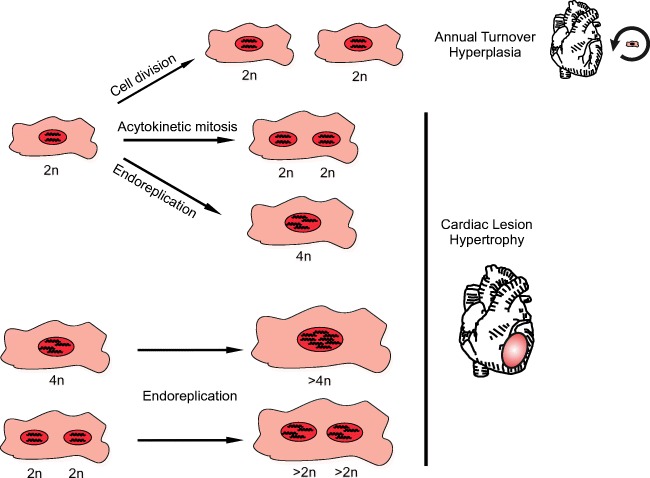
Cardiomyocytes in the mammalian heart switch from hyperplastic to hypertrophic growth during postnatal development. Hyperplastic growth is driven by cell division while hypertrophic growth displays variations in cardiomyocyte cell cycle such as acytokinetic mitosis (karyokinesis, but no cytokinesis), resulting in a binuclear cardiomyocyte and endoreduplication (no karyokinesis or cytokinesis), resulting in a polyploid cardiomyocyte. While evidence is relatively scant, it is believed that diploid, mononuclear cardiomyocytes are still able to divide and are the source of an annual cardiomyocyte turnover. After cardiac injury, however, diploid, mononuclear, polyploid, and/or binuclear cardiomyocytes undergo cell cycle variants and do not fully divide

As there is convincing evidence from ^14^C studies for cardiac turnover, there has to be some source of new cardiomyocytes in the human heart. As there is overwhelming evidence excluding a pool of cardiac stem or progenitor cells, it is likely to be a subpopulation of resident cardiomyocyte. Although it has been shown that binuclear cells can divide too, at least under cell culture conditions with FGF1 stimulation and inhibition of p38-α map kinase, even under those artificial conditions only a minority (~ 6%) displayed authentic cell division [35]. This fact renders binucleated cardiomyocytes unlikely to contribute to turnover leaving mononuclear cardiomyocytes as the most plausible source. Yet, despite the fact that normal cardiomyocyte genesis or replacement is likely to come from existing mononuclear, diploid cardiomyocytes, there is lack of direct evidence for cardiomyocyte hyperplasia after cardiac injury (Fig. 1). However, strategies that aim at augmenting the potential of mononuclear, diploid cardiomyocytes to divide after injury will continue to generate significant interest from cardiovascular investigators. Promising pro-proliferative factors have already been identified [36, 37], but their verification in vivo is still pending. For such studies, it will be critical to use tools that can discriminate between authentic cell division and variants of the cell cycle [33, 38•].
